# Accuracy of Identifying Acute Stroke Admissions in a Michigan Stroke Registry

**Published:** 2011-04-15

**Authors:** Mathew J. Reeves, Susan Wehner, Natalie Organek, Gretchen L. Birbeck, Andrew J. Mullard, Bradley S. Jacobs, Rashmi Kothari, Susan Hickenbottom, for the Paul Coverdell National Acute Stroke Registry Michigan Prototype Investigators

**Affiliations:** Department of Epidemiology, Michigan State University; Michigan State University, East Lansing, Michigan; Michigan State University, East Lansing, Michigan; Michigan State University, East Lansing, Michigan; Michigan State University, East Lansing, Michigan; Wright State University, Boonshoft School of Medicine, Dayton, Ohio; Borgess Health Alliance, Kalamazoo, Michiga; University of Michigan, Ann Arbor, Michigan

## Abstract

**Introduction:**

The accurate identification of acute stroke cases is an essential requirement of hospital-based stroke registries. We determined the accuracy of acute stroke diagnoses in Michigan hospitals participating in a prototype of the Paul Coverdell National Acute Stroke Registry.

**Methods:**

From May through November 2002, registry teams (ie, nurse and physician) from 15 Michigan hospitals prospectively identified all suspect acute stroke admissions and classified them as stroke or nonstroke. Medical chart data were abstracted for a random sample of 120 stroke and 120 nonstroke admissions. A blinded independent physician panel then classified each admission as stroke, nonstroke, or unclassifiable, and the overall accuracy of the registry was determined.

**Results:**

The physician panel reached consensus on 219 (91.3%) of 240 admissions. The panel identified 105 stroke admissions, 93 of which had been identified by the registry teams (sensitivity = 88.6%). The panel identified 114 nonstroke admissions, all of which had been identified as nonstrokes by the registry teams (specificity = 100%). The positive and negative predictive value of the registry teams' designation was 100% and 90.5%, respectively. The registry teams' assessment of stroke subtype agreed with that of the panel in 78.5% of cases. Most discrepancies were related to the distinction between ischemic stroke and transient ischemic attack.

**Conclusion:**

The accuracy of hospitals participating in a hospital-based stroke registry to identify acute stroke admissions was very good; hospitals tended to underreport rather than to overreport stroke admissions. Stroke registries should periodically conduct studies to ensure that the accuracy of case ascertainment is maintained.

## Introduction

The accurate identification of acute stroke admissions is an essential requirement for hospital-based stroke registries; however, the process of accurately distinguishing acute stroke admissions from conditions that mimic stroke requires substantial clinical experience and knowledge (1). Similarly, the accurate identification of stroke subtypes is important for quality improvement–based registries where compliance with performance measures is determined within specific subtypes.

A handful of studies have assessed the reliability or interobserver agreement of the clinical diagnosis of stroke and transient ischemic attack (TIA) (2-5) or have reported on the reliability of clinical stroke classification systems (5-7). However, few reports have assessed the accuracy of clinical stroke diagnosis (8,9), and these studies were conducted in the context of assessing the accuracy of physicians to identify stroke at the first point of medical contact. We could find no studies that have evaluated the accuracy of stroke diagnoses in the context of hospital-based registries or databanks. We therefore conducted a study to determine the ability of Michigan hospital registry teams participating in a prototype of the Paul Coverdell National Acute Stroke Registry to prospectively identify acute stroke cases from among all possible stroke admissions. Our objectives were to determine the accuracy in Michigan hospitals of acute stroke admissions and specific stroke subtypes identified in a statewide stroke registry.

## Methods

### Registry design

The Michigan Acute Stroke Care Overview and Treatment Surveillance System (MASCOTS) was a statewide, hospital-based, acute stroke registry that was a prototype for the Paul Coverdell National Acute Stroke Registry (10,11). Details of the design of the MASCOTS registry have been published previously (12,13). Briefly, MASCOTS used a modified stratified sampling regime as part of a single-stage cluster design to obtain a sample of 16 Michigan hospitals. In the initial pilot phase of the project, 8 large academic-affiliated hospitals from 4 urban communities participating in a community-based stroke project were selected. Another 8 hospitals were then randomly selected from the remaining 114 acute care hospitals according to size (12,13). The MASCOTS registry collected data during  May through November 2002.

### Hospital training, case ascertainment, and stroke subtype designation

All hospital personnel hired for the project attended a group training session before the start of data collection and had access to ongoing training and technical support throughout the data collection period (13). At each hospital, registry teams were defined as consisting of at least 1 nurse, who was either experienced in acute stroke care or was a member of the quality improvement staff, and a physician with interest in stroke who was trained in either neurology or emergency medicine. The stroke physician served as the project's physician champion (ie, the physician who provided central leadership, consultation, and authority for the project within the hospital). Financial support was provided to each hospital so that the lead registry nurse could work on the project between a quarter to half-time, depending on the size of the stroke case load.

Hospital personnel were instructed to identify all acute stroke admissions during May through November 2002 by using prospective case ascertainment methods, defined as the active, systematic, and regular screening of all potential admissions that does not rely on the use of discharge diagnosis codes (14). Each registry team was instructed to regard all consecutive hospital admissions of patients aged 18 or older who had a chief complaint or clinical signs and symptoms consistent with acute stroke as suspected stroke cases and to record them in a MASCOTS logbook. The registry teams identified suspect acute stroke admissions by using "hot pursuit" case ascertainment methods (14), which involved the active and regular review of all data sources that identify potential admissions. These include the admission logs from the emergency department (ED), intensive care unit (ICU), and hospital wards, and neurology consultation logs.

Within 72 hours after the hospital admission, the registry team was instructed to make a judgment about whether a particular suspect stroke case was either a probable acute stroke (defined as a ≥90% probability), not an acute stroke (defined as a <5% probability), or a possible acute stroke (defined as ≥5% to <90% probability. Probable and possible acute stroke cases were then assigned a MASCOTS Study Identification (ID) number on the log book. Cases were included in the final registry if they met 1 of the Coverdell acute stroke subtype case definitions adapted from previous work (15): ischemic stroke (IS), intracerebral hemorrhage (ICH), subarachnoid hemorrhage (SAH), TIA, ischemic stroke of uncertain duration (ISUD), and stroke of uncertain type (11). The final case status was determined by the registry team once the completed medical record became available after the patient was discharged. This determination was made on the basis of detailed review of the Coverdell case definitions (Appendix) and relied heavily on the results of all brain imaging studies and physician and nurse notes.

### Independent hospital audit

As part of the registry's quality assurance efforts (16), a research nurse (S.W.) experienced in acute stroke conducted an independent audit at each registry hospital. The audits were conducted between 6 and 12 weeks after the start of data collection and hospitals received only 2 days' notice of their audit date. Choosing a random starting point in the MASCOTS logbook, the nurse selected 8 sequential admissions that had been designated by the registry team as probable or possible acute stroke cases, and 8 sequential admissions that had been identified as nonstroke cases. Each of the 16 admissions was assigned a unique audit ID number and underwent a full abstraction of the medical chart. The audit nurse had access to the same chart information as the registry team. Clinical information relevant to the diagnostic process (ie, chief complaint, past medical history, ED presentation, imaging results, laboratory and diagnostic test results, consultation summaries, treatments, hospital course, and discharge disposition) were recorded on a separate diagnostic abstraction form. Because 1 of the original 16 hospitals closed shortly after the start of registry enrollment, there were a total of 240 (ie, 16 × 15) completed audit cases. The original sample size of 256 (16 × 16) audit cases was designed to generate overall sensitivity and specificity estimates that were accurate within ±7% (17).

### Review panel and Delphi process

In 2003 an independent panel of 4 physician coinvestigators (3 neurologists and 1 emergency medicine specialist) was assembled to classify each admission as either an acute stroke, nonstroke, or unclassifiable (if stroke status could not be determined). If a case was determined to be an acute stroke admission, panel members then assigned a Coverdell subtype definition by using the case definition criteria (Appendix). A Delphi process was used to facilitate consensus among panel members. The Delphi technique is a structured communication technique used to determine the extent of agreement and to reach consensus among experts (18). Each panel member received a copy of the 240 diagnostic abstraction forms, which were blinded as to the registry team's final designation of stroke or nonstroke. For round 1, panel members recorded their findings on an electronic spreadsheet that was returned to the main study office. Discrepancies in the assignment of acute stroke status and stroke subtype were then identified in an electronic report that documented the nature of the discrepancy and listed the anonymous answers of the 4 reviewers. This electronic report was then shared among the panel members who were then asked to either modify their classification or justify why it should remain the same. Two additional adjudication rounds were undertaken to refine the designation of stroke versus nonstroke and the subtype designation, before holding a final face-to-face meeting. The ultimate aim of this process was to maximize the proportion of cases for which there was complete agreement among the 4 panel members. When the panel could not reach consensus on a case, the case was placed in the unclassifiable group.

Using the panel's final determination as the gold standard, the accuracy of the registry teams' final stroke designation was estimated by calculating the sensitivity (ie, the proportion of acute stroke admissions designated by the panel that were identified by the registry teams) and specificity (ie, the proportion of nonstroke admissions designated by the panel that were identified by the registry teams). Positive and negative predictive values of the registry teams' designations were also calculated. Confidence intervals (CIs) were calculated by using Confidence Interval Analysis software version 2.0.2 (University of Southampton, Southampton, United Kingdom).

For cases in which the physician panel and hospital registry teams agreed that the case represented an acute stroke admission, we assessed the agreement in Coverdell stroke subtypes between the panel and registry teams. Because of the small sample size for some of the subtypes, we were only able to assess the accuracy of the registry teams to identify IS. We used the panel's designation of IS as the gold standard to calculate sensitivity (ie, the proportion of IS admissions designated by the panel that were identified by the registry teams) and specificity (ie, the proportion of non-IS admissions designated by the panel that were identified by the registry teams).

## Results

Of the original 120 probable or possible stroke admissions sampled, 96 (80%) were determined by the registry teams to have met the case definition for an acute stroke admission and were given a final Coverdell stroke subtype diagnosis. The 24 cases not given a subtype diagnosis by the registry teams were considered to be nonstroke admissions and were added to the original 120 nonstroke admissions, making a total of 144. Of the 240 cases reviewed by the physician panel, consensus on whether the case represented an acute stroke admission was reached on 219 (91.3%). Of these, 105 were determined to be acute stroke admissions, and 114 nonstroke admissions. The accuracy of hospital registry teams to identify acute stroke admissions was similar to that of the physician panel (Table 1). The overall sensitivity and specificity of the registry teams to identify acute stroke admissions was very high (88.6% and 100%, respectively) (Table 1). Similarly, the positive and negative predictive values of the registry teams' determinations were also very high (100% and 90.5%, respectively).

The sensitivity of the hospital registry teams to identify acute stroke admissions was not 100% because of 12 cases that were determined by the panel to be acute stroke admissions but were designated as nonstroke admissions by the registry teams. Six of these cases had originally been given a MASCOTS ID number in the logbook (ie, were designated a probable or possible acute stroke). However, this status was changed to nonstroke by the registry team after final review although the clinical presentation and imaging results of all 6 cases appeared to be consistent with acute stroke. The other 6 cases were never given a MASCOTS ID number by the registry team; 3 cases had no clinical symptoms or clinical symptoms that were resolving, 4 had negative imaging findings, and 2 did not have a neurology consult.

The calculations of sensitivity, specificity, and predictive values did not include data on the 21 cases for which the panel could not reach consensus (ie, were designated unclassifiable). Three of these cases were classified as stroke cases by the hospitals, and 18 as nonstroke cases (note that the option to use the unclassifiable designation was not available to the registry teams). After detailed review of these cases, we found that 6 cases had equivocal or resolving clinical symptoms, 19 had initial imaging findings that were negative for an acute or subacute stroke lesion, and only 6 had a definitive determination of stroke or TIA recorded in the chart based on a neurology consult. In 9 cases the original hospital designation of stroke was changed to nonstroke after the patient was discharged.

The physician panel and registry teams generally agreed on the Coverdell stroke subtype diagnosis (Table 2). Agreement on subtype was examined only among the 93 cases for which both the panel and the hospitals agreed were acute stroke admissions. The overall agreement across all stroke subtypes was 78.5% (ie, 73 of 93). Of the 67 cases designated as IS by the panel, the registry teams identified 55, resulting in a sensitivity of 82% (95% CI, 71%-89%). The most common cause of discrepancies in the identification of IS was the difficulty in distinguishing IS from TIA; 10 of the 12 false-negative IS designations were labeled as TIA cases by the registry teams, whereas 5 of the 7 false-positive IS designations were labeled as TIA by the panel. Of the 26 cases designated as non-IS by the panel, the teams correctly identified 18, resulting in a specificity for the designation of non-IS of 69% (95% CI, 50%-84%).

The prevalence of other subtypes was too low to calculate individual sensitivity and specificity estimates. However, overall, the registry teams agreed with the panel's determination in 8 of 9 of the ICH cases, 4 of 6 of the SAH cases, 3 of 8 of the TIA cases, and 3 of 3 of the ISUD cases.

## Discussion

This study found that registry teams participating in a statewide hospital-based acute stroke registry were able to accurately identify suspect stroke admissions, successfully determine those that represented acute stroke cases, and assign an appropriate stroke subtype. We found that hospital registry teams had a tendency to underreport rather than overreport acute stroke admissions. With the exception of 3 cases that the panel could not agree on, we found no evidence of false-positive designations by the hospitals. All acute stroke admissions identified by the registry teams were confirmed by the independent panel (specificity 100%, positive predictive value 100%). The tendency of hospitals to underreport acute stroke admissions was reflected in the 12 false-negative cases, which resulted in a sensitivity of 88.6% (negative predictive value, 90.5%). For the 6 cases in which the original stroke designation was retracted by the hospitals after final review, we found that the clinical presentation and imaging results were all consistent with acute stroke. Thus, the change in designation to nonstroke after discharge appears to represent an error by the registry teams. The initial presentation and work-up of 4 of the other 6 false-negative cases was at least equivocal for an acute stroke admission, which helps explain why the registry teams determined them to be nonstroke admissions.

The lack of comparable studies precludes any meaningful comparison between the findings of this study (in terms of sensitivity and specificity) and other research.  Because this study assessed the final stroke designation after the subject had been discharged from the hospital, its results are not directly comparable to those of the few studies that have evaluated the accuracy of the initial stroke diagnoses obtained at the bedside or by emergency physicians (5-7). Although our data on the accuracy of Coverdell stroke subtype diagnoses were limited by the small numbers of non-IS subtypes, our study was still able to illustrate the widely recognized problem of distinguishing between IS and TIA (19,20). There were 13 cases designated as TIA by the registry teams and 8 cases designated as TIA by the panel, yet in only 3 cases did the registry teams and panel agree. The clinical distinction between the 2 subtypes required the documentation of the duration of symptoms (ie, <24 vs >24 hours), information that was frequently lacking in the medical chart. It should be noted that a new definition of TIA has been proposed (20), which relies on identifying the absence of acute infarction on brain imaging rather than on an arbitrary time period. However, whether this new definition will lead to an improvement in the ability to distinguish between TIA and IS is unknown.

The Delphi approach provided an efficient mechanism that enabled the expert panel to reach consensus on most of the cases they evaluated. We found good reasons why the panel was not able to reach consensus on the status of 21 subjects. Although most cases had a combination of history and clinical findings that was consistent with an acute stroke onset, more than 90% had brain imaging findings that were negative for an acute stroke process. Whereas 5 cases had a definitive stroke diagnosis after neurology consultation (1 IS, 1 TIA, 1 hemorrhagic stroke, and 2 cases of TIA or IS), the panel thought that the evidence presented was not definitive for any of these cases.

One strength of this study is that it tested the accuracy of acute stroke case identification in a stroke registry that had a range of representative hospitals selected by using valid sampling methods (21). The selected hospitals had a range of capabilities and experiences with respect to acute stroke care — varying from large academic medical centers with extensive preexisting stroke capabilities to small rural hospitals with no experience in identifying and tracking acute stroke admissions. Our data show that training a diverse group of hospitals and staff to accurately identify acute stroke admissions is possible. Although the study was not designed to evaluate the performance of individual hospitals, an evaluation of the 16 case abstractions from each hospital did not reveal any outliers in terms of a higher-than-expected error rate at a particular institution. To examine the effect of hospital size and capacity on our findings, we stratified the data into 2 groups of hospitals — the 8 large academic-affiliated hospitals that were involved in the pilot phase of the registry and the remaining 8 hospitals that were selected by stratified sampling methods (this group included medium and smaller hospitals). We found that the sensitivity of stroke designations were the same (ie, 87% in the pilot phase hospitals and 90% in the second randomly selected group). Although the methods used to evaluate the accuracy of this registry are generalizable to other registries and hospitals, our specific findings may not be. As suggested in the context of quality assurance for both epidemiologic studies (4) and disease registries (22), it is prudent to undertake periodic assessments of diagnostic accuracy to confirm that diagnostic biases or misapplication of case definition criteria have not occurred.

Our study also has limitations. First, the audit was conducted during the early phase of the registry (between 6 and 12 weeks of the start) so that any problems with case ascertainment or assignment of acute stroke case definitions could be detected and corrected. Thus, the findings may not be representative of the long-term accuracy of the registry. The accuracy of the registry teams to identify acute stroke admissions and assign stroke subtypes may have changed as more experience was gained during the data collection period. Second, the small number of subjects examined at each hospital precluded the presentation of hospital-specific findings. Similarly, the small number of non-IS subtypes prevented us from calculating sensitivity and specificity estimates for these subtypes. Finally, the only information available to the panel was the diagnostic abstraction form prepared by the research nurse. This information in turn was limited by the quality of the documentation in the medical chart. The panel's determinations may have been different if they had had full access to all of the original information, including imaging studies. However, such an approach was not feasible within the constraints of the study.

Our results illustrate that, with training, a diverse group of hospitals can accurately identify acute stroke admissions by using prospective case ascertainment methods. The assessment of a registry's accuracy and completeness is an essential step and should be undertaken soon after the start of a registry and periodically thereafter to ensure that accurate case ascertainment and case definition are maintained (22,23). We believe this process to be in the context of quality improvement registries, where the potential for bias in the selection of cases into the registry (whether occurring insidiously or otherwise) remains a possibility (24).

## Figures and Tables

**Table 1:** Overall Accuracy of Hospital Registry Teams to Identify Acute Stroke Admissions Relative to That of an Independent Physician Panel (N = 240)^a^

Registry Teams Classification	Independent Physician Panel Classification			

	Stroke	Nonstroke	Unclassifiable	Total

	N = 105	N = 114	N = 21	N = 240
Stroke	93	0	3	96
Nonstroke	12	114	18	144

Abbreviation: CI, confidence interval.

a The independent physician panel was considered the gold standard.Sensitivity = 93/105 = 88.6% (95% CI, 81.1%-93.3%).Specificity = 114/114 = 100% (95% CI, 96.7%-100%).Positive predictive value = 93/93 = 100% (95% CI, 96.0%-100%).Negative predictive value = 114/126 = 90.5% (95% CI, 84.1%-94.5%).

**Table 2:** Agreement of Coverdell Stroke Subtype Diagnosis Between the Independent Physician Panel and Hospital Registry Teams Among Confirmed Acute Stroke Admissions (N = 93)

Registry Teams Classification	Independent Physician Panel Classification				

	IS (N = 67)	ICH (N = 9)	SAH (N = 6)	TIA (N = 8)	ISUD (N = 3)
IS (N = 62)	55	—	2	5	—
ICH (N = 9)	1	8	—	—	—
SAH (N = 5)	—	1	4	—	—
TIA (N = 13)	10	—	—	3	—
ISUD (N = 4)	1	—	—	—	3

Abbreviations: IS, ischemic stroke; ICH, intracerebral hemorrhage; SAH, subarachnoid hemorrhage; TIA, transient ischemic attack; ISUD, ischemic stroke of uncertain duration.

## Appendix. Coverdell Acute Stroke Subtype Case Definitions

**Table:**

**Ischemic stroke (IS):** A rapid onset of focal neurologic deficit with signs or symptoms persisting longer than 24 hours and not attributable to another disease process. Patients who, more than 24 hours after the onset of stroke, have only persistent sensory symptoms with minimal sensory signs or mild impairment of dexterity with normal muscle strength are included. Initial computerized axial tomography/magnetic resonance imaging (CT/MRI) may show evidence of acute IS or no evidence of stroke. When CT/MRI shows an area consistent with intracerebral hemorrhage, this may be seen only in cases with a hemorrhagic transformation of a cerebral infarct.
**Intracerebral hemorrhage (ICH):** Nontraumatic abrupt onset of severe headache, altered level of consciousness and/or focal neurologic deficit that is associated with a focal collection of blood within the brain parenchyma on CT or at autopsy and is not due to trauma or hemorrhagic conversion of a cerebral infarction. Cases of intraventricular hemorrhage without ICH or subarachnoid hemorrhage will be classified as ICH unless angiogram demonstrates an aneurysm.
**Subarachnoid hemorrhage (SAH):** Nontraumatic abrupt onset of severe headache or altered level of consciousness that is associated with blood in the subarachnoid space on CT or at autopsy, or a clinical history and examination consistent with SAH (sudden onset of severe headache or altered level of consciousness) with xanthochromia and many red blood cells in the cerebrospinal fluid. Cases that have both ICH and SAH are classified as SAH if an aneurysmal source of bleeding is documented or if the study investigator suspects a subarachnoid origin of the bleeding. Cases are classified as ICH if a parenchymal source of bleeding seems most likely.
**Transient ischemic attack (TIA):** Patients with acute neurologic signs and symptoms that last less than 24 hours. Patients with transient symptoms that are associated with an appropriate lesion on CT/ MRI will be included as a TIA but not as a case of cerebral infarction. Exclude patient if symptoms no longer exist at presentation to the emergency department.
**Ischemic stroke of uncertain duration (ISUD):** Rapid onset of a major focal neurologic deficit that is consistent with cerebral infarction but the duration of which symptoms could not be confirmed as lasting longer than 24 hours. This category is therefore used when there is insufficient information to distinguish among IS and TIA.
**Stroke of uncertain type:** Rapid onset of a major focal neurologic deficit that persists more than 24 hours or is fatal and cannot be attributed to another cause. This category is used when radiographic or pathologic information is insufficient to distinguish among cerebral infarction, ICH, and SAH.
